# Development of a miRNA-seq based prognostic signature in lung adenocarcinoma

**DOI:** 10.1186/s12885-018-5206-8

**Published:** 2019-01-08

**Authors:** Chathura Siriwardhana, Vedbar S. Khadka, John J. Chen, Youping Deng

**Affiliations:** Bioinformatics and Biostatistics Cores, Department of Complementary and Integrative Medicine, University of Hawaii John A. Burns School of Medicine, Honolulu, HI 96813 USA

**Keywords:** Lung adenocarcinoma, MiRNA, Prognostic signature, Survival

## Abstract

**Background:**

We utilized miRNAs expression and clinical data to develop a prognostic signature for patients with lung adenocarcinoma, with respect to their overall survival, to identify high-risk subjects based on their miRNA genomic profile.

**Methods:**

MiRNA expressions based on miRNA sequencing and clinical data of lung adenocarcinoma patients (*n* = 479) from the Cancer Genome Atlas were randomly partitioned into non-overlapping Model (*n* = 320) and Test (*n* = 159) sets, respectively, for model estimation and validation.

**Results:**

Among the ten miRNAs identified using the univariate Cox analysis, six from miR-8, miR-181, miR-326, miR-375, miR-99a, and miR-10, families showed improvement of the overall survival chance, while two miRNAs from miR-582 and miR-584 families showed a worsening of survival chances. The final prognostic signature was developed with five miRNAs—miR-375, miR-582-3p, miR-326, miR-181c-5p, and miR-99a-5p—utilizing a stepwise variable selection procedure. Using the KEGG pathway analysis, we found potential evidence supporting their significance in multiple cancer pathways, including non-small cell lung cancer. We defined two risk groups with a score calculated using the Cox regression coefficients. The five-year survival rates for the low-risk group was approximately 48.76% (95% CI = (36.15, 63.93)); however, it was as low as 7.50% (95% CI = (2.34, 24.01)) for the high-risk group. Furthermore, we demonstrated the effect of the genomic profile using the miRNA signature, quantifying survival rates for hypothetical subjects in different pathological stages of cancer.

**Conclusions:**

The proposed prognostic signature can be used as a reliable tool for identifying high-risk subjects regarding survival based on their miRNA genomic profile.

**Electronic supplementary material:**

The online version of this article (10.1186/s12885-018-5206-8) contains supplementary material, which is available to authorized users.

## Background

Lung cancer is the leading cause of cancer deaths in the United States (US) and several other developed countries worldwide [1, 2]. In 2017, the number of estimated deaths caused by lung cancer in the US was about 155,870, with 222,500 estimated new cases [2]. Lung cancer is categorized into two major types—non-small cell lung cancer (NSCLC) and small cell lung cancer (SCLC), which account for approximately 85 and 15% of all lung cancers, respectively [3]. NSCLC consists of three main histological types—adenocarcinoma, squamous cell carcinoma, and large cell carcinoma [3]; lung cancer adenocarcinoma is the most common type of NSCLC.

Although early-stage NSCLC patients have been found to be substantially benefited by the surgical approach, about 30–55% of patients develop recurrence and die of the disease despite resection [4]. During the last decade, there has been a significant advancement in personalized target therapeutic strategies for cancers, including lung cancer [5, 6], showing the need of exploring genomic subgrouping for selecting optimal treatment, instead of following a “one size fits all” concept. Particularly, lung cancer is now widely reorganized as a heterogeneous disease and patients are often subdivided into molecular subtypes with targeted chemotherapeutic strategies [5, 7]. For adenocarcinoma, two types of strategies—known as tyrosine kinase inhibitor, for tumors carrying mutations in the tyrosine kinase domain of epidermal growth factor receptor (EGFR), and crizotinib for tumors with the rearrangement of anaplastic lymphoma kinase (ALK)—are currently available as commercial personalized treatments [5, 7].

MicroRNAs (miRNAs) are small, non-coding RNA molecules, about 17–25 nucleotides in length, which critically influence on a wide range of biological processes through controlling messenger RNA by either degradation or repressing translation into protein [8]. In recent years, the role of microRNA in cancers has been widely investigated; several studies have shown the importance of miRNAs as both oncogenes and tumor suppressors by regulating cell proliferation, cell adhesion, apoptosis, and angiogenesis [9]. Several studies have discussed survival characteristics with respect to miRNA profiles, including NSCLC [9, 10], demonstrating miRNAs as promising diagnostic, prognostic, and predictive biomarkers for cancer studies. Furthermore, studies have demonstrated the long-term stability of miRNAs in formalin-fixed, paraffin embedded tissues for different cancer types [11]. Additionally, its stability in human plasma shows the capability of extracting miRNA expressions from body fluids, the “liquid biopsies”, including blood [12] and urine [13] for identifying biomarkers using non-invasive techniques.

Prognostic biomarkers are individual-specific characteristics such as genomic indicators including mRNAs, miRNAs, or genes themselves that predict a certain clinical or quality of life outcome. Particularly for cancers, the most common outcome is survival. In the last two decades, with rapid advances in genomic research, there have been numerous efforts to develop prognostic signatures for multiple cancers [14, 15], including lung adenocarcinoma [16, 17], to formulate genomic tests for target treatment selection. To our knowledge, there exist few reports using miRNA sequencing data from TCGA to identify a prognostic signature for lung adenocarcinoma; these studies report diverse findings. We believe that further studies should be conducted in this field. Therefore, in this work, we have developed a prognostic signature using multiple miRNAs for lung adenocarcinoma with respect to the individuals’ overall survival, which can potentially be used as a tool for identifying high-risk subjects with an increased risk for mortality.

## Methods

### Data

The Cancer Genome Atlas (TCGA) lung adenocarcinoma data were obtained from OmicSoft OncoLand (OmicSoft Corp; Cary, NC). We obtained miRNA expressions measured in tumor tissues of lung adenocarcinoma patients based on the next-generation sequencing technology and their clinical and non-clinical data describing several characteristics including demographics, smoking history, disease stages, and overall survival times. The data contained approximately 2800 miRNA expressions measured in tissues from 479 subjects. Since most miRNAs contained excessive zeros in their expression levels, a reduced set of 672 miRNAs were used; these contained 25% or lesser zeros in their expressions and were selected for the current analysis. The miRNAs with more than 25% zero expression measurements, approximately 99% of cases in the 75th percentile of miRNA expressions, was less than or equal to 1.

Among 479 lung adenocarcinoma patients, 261 (54.5%) were diagnosed with Stage 1 disease, 118 (24.6%) with Stage 2, 76 (15.9%) with Stage 3, and 24 (5%) with Stage 4 (Table 1). Most subjects were whites (78%). Approximately 55.5% were females. The patients’ overall survival times were widely subjected to right censoring, resulting in an approximately 80% censoring rate.

**Table 1:** Clinical characteristics of lung adenocarcinoma patients

Num. of patients	479
Age in years, mean (SD)	65.39 (9.93)
Females, count (%)	261 (54.48)
Ethnicity, count (%)	
Asian	8 (1.67)
African American	47 (9.81)
White	378 (78.91)
Other/Unknown	46 (9.60)
Cancer Stage, count (%)	
Stage-1	261 (54.49)
Stage-2	118 (24.63)
Stage-3	76 (15.87)
Stage-4	24 (5.01)
Smoking History, count (%)	331 (69.10)

### Model/test set

479 subjects from the original dataset were randomly divided into two non-overlapping sets—“Model” and “Test”—using simple random sampling, selecting 320 for the Model set (i.e., training set) and 159 for the Test set (i.e., validation set). This group assignment provides approximately 67 and 33% allocation rates to the Model and Test sets, respectively.

### Statistical methods

The effect of miRNAs on the overall survival of lung cancer was primarily evaluated using univariate and multivariable Cox proportional hazard model approaches. For the stability of the Cox regression coefficient estimates, we used *log* transformations of observed miRNA expression measurements. Expressions were initially truncated at the lower threshold at 0.05 to facilitate transformation.

Using the Model set, the effect of each 672 miRNAs on the overall survival was evaluated based on a set of independent univariate Cox proportional hazard models, assigning the dependent variable to be right-censored survival times. Observed *p*-values for miRNAs were next adjusted for the False Discovery Rate (FDR) to eliminate false significance by random chance. An upper threshold of FDR adjusted p-value 0.05 was used to find a suitable set of miRNAs that can be potentially included in a multivariable Cox model. A variable selection procedure developed on minimizing the Akaike Information Criterion (AIC) by adding and dropping was utilized to find a reduced set of miRNAs.

Hereafter, to filter-out the effects of additional risk factors, a set of demographic and clinical risk factors were included to estimate a final proportional hazard model. Such adjustment allows one to extract the accurate effects of each miRNA on survival hazard. The analysis of deviance was conducted, comparing the model containing clinical and risk factors alone to the one integrating miRNAs as predictors, to see the overall significant contribution of miRNAs on the hazard modeling. The signs of the estimated regression coefficients of selected miRNAs were examined to classify those that either improve or worsen the survival of a cancer patient. The proportional hazard assumption of model covariates was tested for the validity of the Cox regression model. This study was conducted in *R* − 3.3.1 base software with several supporting *R* packages.

### Risk score

A risk score for each subject in the Model set was calculated by taking into account the linear combination of miRNAs and additional risk factor effects based on estimated regression parameters and observed values. The score value *S*_i_(*X*_i_, *Z*_i_) for the *i*th, *i* = 1,..,*n*, subject is given by the following:

1 $$ {S}_i\left({X}_i,{Z}_i\right)=\sum \limits_{j=1}^J{\hat{\beta}}_j\kern0.5em \times \kern0.5em {X}_{ij}+\kern0.5em \sum \limits_{k=1}^K{\hat{\gamma}}_k\kern0.5em \times \kern0.5em {Z}_{ik} $$

Where, ${\widehat{\beta}}_j,{\widehat{\gamma}}_k$ are coefficients estimated for *X*_*j*_ th miRNA and *Z*_*k*_ th additional risk factor, with *j* = 1,.., *J*; *k* = 1,.., *K*, and *X*_*i*_ = {*X*_*i*1_, …, *X*_*iJ*_}, *Z*_*i*_ = {*Z*_*i*1_, …, *Z*_*iK*_}. Note, that higher score values indicate the increased hazard of death by cancer, at any given time. A threshold value was specified as the 60% quantile point of the calculated scores for classifying subjects into high-risk and low-risk groups. Although this is an ad-hoc threshold, similar criteria—such as percentile points such as 75%—has been used in the literature [17]. Suppose *Q*_60_ is the 60%th percentile of the estimated scores. The high-risk group *R*_*i*_(*X*_*i*_, *Z*_*i*_) would then be defined as the following:

2 $$ {R}_i\left({X}_i,{Z}_i\right)=\kern0.5em I\left[{S}_i\left({X}_i,{Z}_i\right)\kern0.5em >\kern0.5em {Q}_{60}\right] $$

Overall survival of high-risk and low-risk groups was inspected based on survival curves generated by the Kaplan Meier product-limit estimates, separately for the Model and Test sets. The log-rank test was used to determine the overall difference on the survival hazards between high- and low-risk groups.

### Validation of selected miRNAs

The accuracy of the proposed risk scoring scheme accounting the additive effects of miRNAs and other risk factors was formally evaluated with the Test set. First, a score value for each individual in the Test set was determined using formula-1. Next, the criteria given in formula-2 was used to classifying the individual into a risk group. The Kaplan-Meier survival functions were estimated for high- and low-risk test patients; the statistical significance between the overall hazards of the two groups was determined by the log-rank test.

## Results

### MiRNA association with the survival from the cancer

Table 2 provides a list of 29 miRNAs that had FDR adjusted *p*-values less than 0.2 level when the univariate Cox models were estimated.

**Table 2:** A list of top 29 miRNAs found to be having p-values less than 0.2, after adjusting the originally calculated *p*-value of the univariate Cox proportional hazard models, for FDR. miRNAs were ranked (i.e., top to bottom) based on their *p*-values. The analysis was conducted using the Model set

Rank	miRNA	Cox Reg. Coefficient	*P*-value	*P*-value FDR Adjusted	miRNA Family
1	miR-181c-5p	−1.142	<.0001	0.0001	miR-181
2	miR-200b-3p	−0.604	<.0001	0.0049	miR-8
3	miR-200a-3p	−0.498	<.0001	0.0049	miR-8
4	miR-375	−0.358	<.0001	0.0049	miR-375
5	miR-582-3p	0.352	0.0001	0.0147	miR-582
6	miR-181c-3p	−0.813	0.0001	0.0147	miR-181
7	miR-200b-5p	−0.550	0.0002	0.0211	miR-8
8	miR-99a-5p	−0.537	0.0003	0.0211	miR-10
9	miR-200a-5p	−0.536	0.0006	0.0349	miR-8
10	miR-429	−0.448	0.0006	0.0349	miR-8
11	miR-584-5p	0.398	0.0006	0.0349	miR-584
12	miR-326	−0.466	0.0005	0.0349	miR-326
13	miR-29c-3p	−0.541	0.0010	0.0525	miR-29
14	miR-101-3p	−0.820	0.0016	0.0714	miR-101
15	miR-101-3p 2	−0.823	0.0016	0.0714	miR-101
16	miR-30d-5p	−0.642	0.0017	0.0716	miR-30
17	miR-3065-3p	−0.351	0.0021	0.0820	miR-3065
18	miR-30b-5p	−0.500	0.0032	0.1191	miR-30
19	miR-548b-3p	−0.239	0.0036	0.1196	miR-548
20	miR-30d-3p	−0.558	0.0035	0.1196	miR-30
21	miR-181a-5p	−0.628	0.0050	0.1455	mir-181
22	miR-30b-3p	−0.366	0.0048	0.1455	miR-30
23	miR-181a-5p 2	−0.628	0.0050	0.1455	miR-181
24	miR-582-5p	0.276	0.0052	0.1467	miR-582
25	miR-181d-5p	−0.484	0.0057	0.1540	miR-181
26	miR-491-5p	−0.306	0.0064	0.1651	miR-491
27	miR-29b-2-5p	−0.433	0.0079	0.1822	miR-29
28	miR-3934-3p	−0.273	0.0078	0.1822	mir-3934
29	miR-532-5p	−0.557	0.0079	0.1822	miR-532

FDR = False discovery rate; Cox Reg. = Cox Regression; miRNA = microRNA

A set of twelve miRNAs found significantly affect the overall survival hazard at 5% FDR were chosen for the next stage of analysis. From this set, miRNAs from miR-8, miR-181, miR-326, miR-375, miR-99a, and miR-10, showed negative impacts on the hazard rates with respect to an increase in their expressions. Two miRNAs, mir-582 and mir-584, increased the hazard of death. Five miRNAs—miR-375, miR-582-3p, miR-326, miR-181c-5p, and miR-99a-5p—were finally selected based on the variable selection concept. We examined the effect of clinical and demographic variables available of the patients to identify additional risk factors for hazard modeling. Among several variables available, the pathological state and age variables were found significant. Here, we have categorized the pathological state as Stage-1, Stage-2, and Stage 3, and above (Stage-3+). Note that, due to the inadequate number of subjects reported at Stage-4, the group Stage-3+ was developed by combining the subjects of Stages 3 and 4. Demographic variables such as race/ethnicity (likelihood ratio *p*-value = 0.26) and gender (likelihood ratio p-value = 0.58) did not show statistical significances at 5% when the Cox regression models were developed for the corresponding variable. Besides, we did not find a significant effect of the patients’ smoking history on survival (likelihood ratio p-value = 0.93). A multivariate Cox regression model was fitted with five miRNAs while adjusting for the effects of pathological state and age variables. Table 3 shows the estimated regression coefficients and corresponding *p*-values. Based on an analysis of deviance conducted to compare the model containing age and pathological state with the combined model including the effects of miRNAs, we found significant improvement by miRNAs on hazard estimation, showing a *p*-value < 0.001 for the likelihood ratio test. Furthermore, we performed the analysis of deviance by adding each of the five selected miRNAs separately to the model that contained only age and pathological states, which produce a likelihood ratio test *p*-value < 0.001, in each case. Moreover, we tested the interaction between Cancer Stage and miRNA on the hazard; such interaction terms were broadly insignificant. None of the model components were found to be violating the proportional hazard assumption. The effect of miR-326 (*p*-value = 0.15) was found not significant at the 5% level. As indicated in Table 4, we did not find a substantial Pearson’s correlation coefficient among any pair of the above five miRNAs.

**Table 3:** Estimated Cox regression coefficients of a set of five miRNAs selected from the 12 miRNAs, via the stepwise variable selection method

		Cox Regression Coefficient	*P*-value
miRNAs	miR-375	−0.3177	0.0009
	miR-582-3p	0.2082	0.0181
	miR-326	−0.2168	0.1506
	miR-181c-5p	−0.6387	0.0080
	miR-99a-5p	−0.4665	0.0092
Other Risk Factors	Age	0.0176	0.1338
	Stage 2 vs. 1	0.7808	0.0064
	Stage 3+ vs. 1	1.5006	<.0001

The proportional hazard model was estimated by incorporating the effects of subjects’ age and pathological stage variables. The analysis was conducted using the Model set

**Table 4:** Pearson’s correlation coefficient estimated for expressions (log transformed), between each pairs of five miRNAs: miR-375, miR-582-3p, miR-326, miR-181c-5p, and miR-99a-5p

	miR-582-3p	miR-326	miR-181c-5p	miR-99a-5p
miR-375	−0.01	0.25	0.28	0.04
miR-582-3p		0.03	0.01	−0.07
miR-326			0.29	0.17
miR-181c-5p				0.35

Increments in the expressions of four miRNAs: miR-375 (HR = 0.74, 95% CI = (0.62, 0.87)), miR-326 (HR = 0.81, 95% CI = (0.60, 1.08)), miR-181c-5p (HR = 0.53, 95% CI = (0.33, 0.85)), and miR-99a-5p (HR = 0.63, 95% CI = (0.44, 0.89)), found to be improving the survival of subjects, while miR-582-3p (HR = 1.23, 95% CI = (1.06, 1.43)) was found to be worsening the survival outcome.

As expected, age factor increased the hazard of death (HR = 1.02 per unit increment, 95% CI = (0.99, 1.04)); however, this was not statistically significant (*p*-value = 0.13) at 5%. Hazards were found to be strongly increasing when moving from Stage-1 to subsequent higher pathological stages: Stage 2 vs. Stage 1 (HR = 2.18, 95% CI = (1.25, 3.83)), and Stage 3+ vs. Stage 1 (HR = 4.48, 95% CI = (2.48, 8.10)).

To confirm our findings, we conducted a KEGG pathway analysis with the selected set of five miRNAs using the DIANA-miRPath v3.0 tool (i.e., using the default settings) [18]. The Additional file 1: Table-S1 shows the results of the analysis. We found forty-three different pathways significantly associated with the selected set of miRNA biomarkers. These were significantly associated with NSCLCs (*p*-value = 0.0177), targeting 17 genes. We present this list of genes in Table 5. Clearly, these miRNAs were found to be strongly associated with multiple cancer pathways, including SCLCs.

**Table 5:** Experimentally supported interactions between five miRNAs: miR-375, miR-582-3p, miR-326, miR-181c-5p, and miR-99a-5p, with 17 genes that are associated with non-small cell lung cancer, using KEGG pathway analysis

Gene	miRNAs
PRKCA	miR-375, miR-582-3p
E2F1	miR-326
ERBB2	miR-375, miR-326
E2F2	miR-326
PIK3CB	miR-181c-5p, miR-99a-5p
RAF1	miR-375, miR-326
EGFR	miR-181c-5p
KRAS	miR-181c-5p
CDK6	miR-99a-5p, miR-582-3p
PIK3R3	miR-181c-5p
CCND1	miR-99a-5p, miR-326
E2F3	miR-582-3p
RB1	miR-181c-5p
AKT3	miR-181c-5p, miR-326
FOXO3	miR-375
MAP2K1	miR-181c-5p
GRB2	miR-181c-5p

### Risk scores and risk groups

**Fig. 1:**
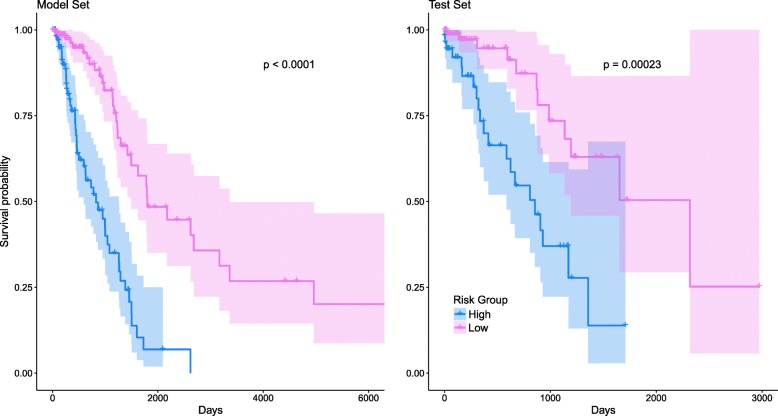
Estimated survival curves for high and low risk groups, separately using Model and Test sets. The p-value provided corresponds to the log-rank test. The colored region represents the 95% point-wise CIs

The risk score for a lung-cancer patient was calculated using formula-1. The threshold score value to specify high- and low-risk groups was determined as *Q*_60_ =  − 5.59. Figure 1 shows the estimated Kaplan Meier survival curves for high- and low-risk groups, using the Model and Test sets.

The *p*-value of the log-rank test conducted for the Model set was < 0.001; agreeing with this, a *p*-value < 0.001 was observed for the Test set. This outcome validated the scoring mechanism that comes from potential biomarker miRNAs and other risk factors. In the supplementary material, we have provided survival curves for high- and low-risk groups (see Additional file 1: Figure S1), obtained using the entire dataset (i.e., combining both Model and Test sets). For both groups, the estimated median survival times were 2.26 years (95% CI = (1.72, 2.96)) and 4.93 years (95% CI = (4.42, 8.68)), respectively. The estimated five-year survival rates for the low-risk group was about 48.76% (95% CI = (36.15, 63.93)), whereas, this was low at 7.50% (95% CI = (2.34, 24.01)) for the high-risk group.

### Illustrating the effect of miRNA profile

Among the remaining five miRNAs in the final model, miR-375, miR-326, miR-181c-5p, and miR-99a-5p were found be improving survival, while miR-582-3p was reducing survival. Suppose ${}{}^S{Q}_j^{25}$ and ${}{}^S{Q}_j^{75}$ are the 25%th and 75%th percentiles expressions levels for the miRNAs *j*, *j*∈ {miR-375, miR-326, miR-181c-5p, miR-99a-5p, miR-582-3p}, for the pathological stage *s*, *s*∈{Stage-1, Stage-2, Stage 3+}. Suppose, the five-dimensional vector ^*s*^*X* contains expression of miR-375, miR-326, miR-181c-5p, miR-99a-5p, and miR-582-3p, respectively for a given subject at stage *s*. We consider two hypothetical individuals of 60 years who, at stage *s*, has expression sets ${{}{}^SX}_{Case-1}$ and ${{}{}^SX}_{Case-2}$ as follows,

$$ {{}^sX}_{Case-1}=\kern0.5em \left({{}^sQ}_{miR-375}^{75},\kern0.5em {{}^sQ}_{miR-326}^{75},\kern0.5em {{}^sQ}_{miR-181c-5p}^{75},\kern0.5em {{}^sQ}_{miR-99a-5p}^{75},\kern0.5em {{}^sQ}_{miR-582-3p}^{25}\right) $$

and

$$ {{}^sX}_{Case-2}=\kern0.5em \left({{}^sQ}_{miR-375}^{25},\kern0.5em {{}^sQ}_{miR-326}^{25},\kern0.5em {{}^sQ}_{miR-181c-5p}^{25},\kern0.5em {{}^sQ}_{miR-99a-5p}^{25},\kern0.5em {{}^sQ}_{miR-582-3p}^{75}\right) $$

Here, ^*s*^*X*_*Case* − 1_ represents an individual with a safe genomic profile, who has higher expression values for miRNAs that improve the survival and lower values for the miRNA that decreases the survival. Alternately, ^*s*^*X*_*Case* − 2_ corresponds to a subject with an increased risk due to their miRNA profile.

**Fig. 2:**
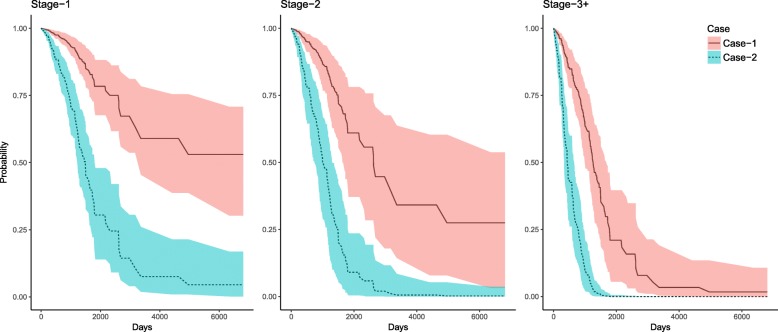
Survival functions estimated for two hypothetical individuals of age 60 with miRNA profiles represent by ^*S*^*X*_*Case* − 1_ and ^*S*^*X*_*Case* − 2_, for given pathological state *s*, *s*∈{State-1, State-2, State 3+}. Four miRNAs: miR-375, miR-326, miR-181c-5p, and miR-99a-5p, are upregulated in Case-1, but downregulated in Case-2. miR-582-3p is downregulated in Case-1, but upregulated in Case-2*.* Survival functions are displayed for *s* = Stage-1, *s* = Stage-2, and *s* = Stage = 3+, scenarios. The colored region represents 95% bootstrap based point-wise CIs

Based on the estimated Cox models’ coefficients, we obtained hazard functions with respect to time using the Breslow estimator for two scenarios (i.e., Case-1 and Case-2). Survival curves were estimated for the two genomic profiles. We developed 95% point-wise CIs for two cases, based on the bootstrap method, using 500 bootstrap samples. Figure 2 provides estimated survival functions along with 95% CI. Note, the above study was conducted using the entire data (i.e., including Model and Test sets), for the reliable approximation of estimates and CIs.

This analysis showed that for an individual aged 60 with a safe profile (i.e., Case-1), the five-year overall survival probabilities are approximately 78.41% (95% CI = (67.66, 88.22)), 60.97% (95% CI = (37.13, 78.14)), and 21.01% (95% CI = (5.47, 39.58)), if the individual is at Stage-1, 2, and 3+, respectively; however, corresponding survival probabilities decrease to 30.47% (95% CI = (14.00, 47.98)), 9.03% (95% CI = (0.41, 23.5)), and 0.03% (95% CI = (<.01, 0.49)), if the subject has a risky profile.

## Discussion

In recent years, with the contribution of rapid development in sequencing technology and genomic research, the reguatory role of miRNAs in biological systems have been widely investigated. Especially, miRNAs’ role in cancer has become an important topic in biomarker studies. With recent advancements, we are moving to a new era of personalized target therapies. Lung cancer is the leading cause of cancer deaths in the US and many other countries worldwide, with NSCLC as the common histology of lung adenocarcinoma [1, 2]. It is thus important to develop genomic tests for lung adenocarcinoma, to predict the survival of a patient based on their individualized features. Additionally, such a tool may potentially be useful in deciding how well a certain target treatment would be beneficial to the patient, referring to their genomic profile. Therefore, in this study, we have developed a prognostic signature for lung adenocarcinoma cancer patients based on miRNAs, focusing on their overall survival.

We have identified a set of potential prognostic biomarker miRNAs for lung adenocarcinoma, examining their effects on the overall survival outcome, and defined high- and low-risk patients using a scoring mechanism accounting for the effects of a set of five miRNAs and other risk factors, such as cancer stage and age. The selection of miRNAs and the proposed scoring criterion was validated using an independent validation dataset. Quantifying the marginal survival characteristics, we have observed a wide difference between both groups. Furthermore, we have provided an illustrative example to demonstrate the impact of the subjects’ genomic profile on five-year survival probability. Among the five miRNAs selected for the multiple Cox regression models—miR-375, miR-582-3p, miR-326, miR-181c-5p, and miR-99a-5p—except miR-582-3p, all others have demonstrated a negative effect on the hazard rate function, when increasing expression levels.

Recently, Wang et al. [19] used seven miRNAs (miR-148b, miR-365, miR-32, miR-375, miR-21, miR-125b, miR-155) to develop a prognostic signature for NSCLC patients on their overall survival outcome. Similar to the signature presented by us, they used miR-375, which is common. Their study used the TCGA miRNA Sequencing data of NSCLC patients (195-lung adenocarcinoma, 145-lung squamous cell carcinoma) and miRNA microarray data from another source. Parallel to our study, Li et al. [20] developed a prognostic signature including miRNA-375 and seven other miRNAs (miR-31, miR-196b, miR-766, miR-519a-1, miR-187, miR-331, miR-101-1). They incorporated the smoking factor in their signature, which we found to be insignificant on survival. Maemura et al. [21] compared lung adenocarcinoma and the corresponding normal counterpart specimen in silico and found expression differences in five miRNAs (miR-379-5p, miR-99a-5p, and miR-497-5p, and miR-200b-3p). They validated the negative association of miR-99a-5p expression and overall survival using the TCGA data. We also have incorporated miR-99a-5p in our prognostic signature. Note that, we found miR-200b-3p to be a top miRNA via univariate Cox analysis; however, it was omitted at the multivariable model fitting stage. Li et al. [22] developed a TCGA data based prognostic signature with four miRNAs (miR-101-1, miR-200a, miR-4661, miR-450a-2). Although none of these miRNAs are presented in our tool, both miR-101-1 and miR-200a were listed in our top list by the univariate Cox models. Another signature developed by Lin et al. [23] based on TCGA data used four miRNAs (miR-148a-5p, miR-31-5p, miR-548v, miR-550a-5p). Their signature does not share any miRNA with ours. Notwithstanding, we should highlight Lin et al.’s [23] study did not account for patient age and cancer stage. Besides, their study did not use a validation step for the confirmation of their findings. In a different study, Sathipati and Ho [24] presented a prognostic signature using eighteen miRNAs (let-7f-1, miR-16-1, miR-152, miR-217, miR-18a, miR-193b, miR-3136, let-7 g, miR-155, miR-3199-1, miR-219-2, miR-1254, miR-1291, miR-192, miR-3653, miR-3934, miR-342, and miR-141), based on optimized support vector regression approach. However, their signature does not share any common miRNAs with our tool. It is important to note that the initial sets of miRNAs used in these method appared to be different, making them incomparable.

The down regulation of miR-375 was found to be associated with an increased hazard of NSCLC patients by multiple studies [19, 20, 25]. Several studies reported the association with down regulation of miR-375 expression with many other cancers, including cervical [26], colorectal [27], and bladder [28]. It has been discovered that miR-375 regulates PDK1 enzyme, involving glucose regulation of insulin gene expression and beta-cell growth [29]; PDK-1 is a potential target for cancer therapy which maintains cell proliferation, survival, nutrients uptake, and storage, by activating its downstream AGC family of protein kinases involved in signaling a complex network system [30]. Furthermore, miRNA-375 inhibits tumor growth and metastasis through repressing IGF-1R that has been identified as a potential therapeutic target for NSCLC patients [31, 32].

We have found that the increased expressions of miR-582-3p are negatively associated with survival. Previous studies show that miR-582-3p expands the cancer stem cell population in NSCLC tissues and negatively associates with both the overall survival and recurrence-free-survival of patients [33]. Additionally, it has been reported that miR-582-3p is associated with colon cancer [34] and Hodgkin lymphoma [35]. Three genes, AXIN2, DKK3, and SFRP1, which are known as tumor suppressors in a broad range of human malignancies including NSCLC [36], are targeted by miR-582-3p and suppress their protein levels [34]. However, it has been found that the increased expression of miR-582-3p reduces the proliferation and invasion of bladder cancer [37]. Based on these findings, miR-582-3p appears to have a dual role as both a tumor suppressor and promoter.

The tumor suppressor function of miR-326 has been reported in various cancers, including lung adenocarcinoma [38, 39]. miR-326 has been found to be suppressing the MRP-1 protein, which is well known for its multi drug resistance action [40]. miR-326 has also been found to be suppressing the oncogene CCND1 expression levels [38].

Two members of the miR-181 family (i.e., miR-181c-5p and mir-181c-3p) have showed strong effects on the hazard (FDR adj. *p*-value < 5%) based on the univariate analysis; however, only miR-181c-5p was selected to the multivariable model when the variable selection was performed. Down regulated expression levels of the miR-181 family has been frequently reported in lung adenocarcinoma tissues [41, 42]. Additionally, the aberrant expression level of this miRNA is reported in many other cancer types including neuroblastoma and glioblastoma [43, 44], indicating it as a tumor suppressor. It is thus evident that the downregulation of this family of expressions is closely associated with the upregulation of BCL2 proteins [42], which plays a critical role in regulating major types of cell death, contributing to cancer development and progression [45].

Previous studies have shown the downregulation of miR-99a-5in NSCLC cells promotes proliferation, migration, and invasion by modulating IGF-1R signaling [46]. Furthermore, it has been suggested that the downregulation of miR-99a-5p promotes cancer progression in human oral carcinoma cells, signaling via NOX4 [47].

There are several limitations within this study. Out of approximately 2800 miRNAs, we have used a subset of 672—those that contained 25% or below 0 s in their individual expression levels for the analysis. As we have described in the Methods Section, sufficient non-zero counts were not available for a large set of miRNAs for a meaningful analysis. In fact, use of miRNAs with largely inflated with 0 s result in technical difficulties in model estimations (e.g., convergence issues due to the lack of variability). The threshold score point (*Q*_60_) for specifying high and low risk groups was an ad-hoc selection and the optimal threshold selection was not a focus in this article. The proposed tool was entirely based on the TCGA sequencing data. Hence, not validating this signature using a different source of data can be considered as another limitation.

Despite these limitations, we believe our findings have the potential utility for predicting the survival chance of a lung adenocarcinoma patient. Interestingly, previous studies have also identified the miRNAs that we recognized as influential on lung adenocarcinoma prognosis, for multiple cancer types. Further investigations with larger numbers of patient samples are appropriate to validate the application of using these biomarkers and the proposed scoring mechanism.

## Conclusion

We developed a prognostic signature using the expression levels of five miRNAs—miR-375, miR-582-3p, miR-326, miR-181c-5p, miR-99a-5p, and miR-582-3p—and the patients’ disease stage and age, to predict the overall survival of lung adenocarcinoma. The proposed signature can be successfully used as a genomic tool to identify high-risk and low-risk patients, based on the proposed risk scoring scheme.

## Additional file

Additional file 1: **Table S1.** A list of KEGG pathways found as significantly associated with five miR-NAs: miR-375, miR-582-3p, miR-326, miR-181c-5p, and miR-99a-5p. The analysis was performed using DIANA-miRPath v3.0 [18]. **Figure S1.** Estimated survival curves for high and low risk groups based on the entire dataset (i.e., combining both Model and Test sets). The p-value provided correspond to the log-rank test. The colored region represents the 95% point-wise CIs. **Figure S2. **A histogram produced for the distribution of p-values that were calculated for the likelihood ratio test, which compares the Cox regression model that contained only cancer stage and age effects versus the model contained the effects of five miRNAs (miR-375, miR-562-3p, miR-326, miR-181-5p, and miR-99a-5p), in addition to cancer stage, and age. P-values were obtained using 1000 randomly selected folds of size n=320 of the original data. **Figure S3.** Histograms produced for distributions of p-values that were calculated for the likelihood ratio test, which compares the Cox regression model that contained only cancer stage and age effects versus models contained effects of the each five individual miRNAs (miR-375, miR-562-3p, miR-326, miR-181-5p, and miR-99a-5p), in addition to cancer stage, and age. P-values were obtained using 1000 randomly selected folds of size n=320 of the original data. (PDF 62 kb)

## Abbreviations

AEG1: Astrocyte Elevated Gene-1
AIC: Akaike information criterion
ALK: anaplastic lymphoma kinase
CI: confidence interval
EGFR: Epidermal growth factor receptor
FDR: False Discovery Rate
HR: hazard ratio
MiRNA: microRNA
MRP1: Multidrug resistance-associated protein 1
NOX4: NADPH Oxidase 4
NSCLC: non-small cell lung cancer
PDK1: Phosphoinositide-dependent kinase-1
SCLC: small cell lung cancer
TCGA: The Cancer Genome Atlas
YAP1: Yes Associated Protein 1
ZEB1: Zinc finger E-box-binding homeobox 1
ZEB2: Zinc finger E-box-binding homeobox 2
