# Supplementary material for: Development of a miRNA-seq based prognostic signature in lung adenocarcinoma
Source: BMC Cancer. 2019 Jan 8;19:34. doi: 10.1186/s12885-018-5206-8 (PMC6325795; doi:10.1186/s12885-018-5206-8)
Supplement: Supplementary file 1 — Table S1. A list of KEGG pathways found as significantly associated with five miR-NAs: miR-375, miR-582-3p, miR-326, miR-181c-5p, and miR-99a-5p. The analysis was performed using DIANA-miRPath v3.0 [18]. Figure S1. Estimated survival curves for high and low risk groups based on the entire dataset (i.e., combining both Model and Test sets). The p-value provided correspond to the log-rank test. The colored region represents the 95% point-wise CIs. Figure S2. A histogram produced for the distribution of p-values that were calculated for the likelihood ratio test, which compares the Cox regression model that contained only cancer stage and age effects versus the model contained the effects of five miRNAs (miR-375, miR-562-3p, miR-326, miR-181-5p, and miR-99a-5p), in addition to cancer stage, and age. P-values were obtained using 1000 randomly selected folds of size n=320 of the original data. Figure S3. Histograms produced for distributions of p-values that were calculated for the likelihood ratio test, which compares the Cox regression model that contained only cancer stage and age effects versus models contained effects of the each five individual miRNAs (miR-375, miR-562-3p, miR-326, miR-181-5p, and miR-99a-5p), in addition to cancer stage, and age. P-values were obtained using 1000 randomly selected folds of size n=320 of the original data. (PDF 62 kb) [file 12885_2018_5206_MOESM1_ESM.pdf]

Supplementary Material

KEGG pathway	p-value	Number of Genes
Proteoglycans in cancer	<0.00001	61
Hippo signaling pathway	<0.00001	43
Protein processing in endoplasmic reticulum	<0.00001	52
Glioma	<0.00001	24
Lysine degradation	<0.00001	15
Adherens junction	<0.00001	25
Prostate cancer	0.00011	34
Renal cell carcinoma	0.00022	24
Pathways in cancer	0.00032	93
Transcriptional misregulation in cancer	0.00035	46
FoxO signaling pathway	0.00044	41
RNA transport	0.00062	48
Colorectal cancer	0.00062	22
Pancreatic cancer	0.00100	24
Hepatitis B	0.00100	42
Viral carcinogenesis	0.00100	50
p53 signaling pathway	0.00158	23
Bacterial invasion of epithelial cells	0.00171	21
mRNA surveillance pathway	0.00238	29
ErbB signaling pathway	0.00509	24
Bladder cancer	0.00509	16
Endocytosis	0.00533	49
Estrogen signaling pathway	0.00551	29
Choline metabolism in cancer	0.00864	32
Shigellosis	0.01418	19
Focal adhesion	0.01469	52
Small cell lung cancer	0.01604	25
Thyroid hormone signaling pathway	0.01617	33
Non-small cell lung cancer	0.01617	16
mTOR signaling pathway	0.01698	19
Salmonella infection	0.01698	24
PI3K-Akt signaling pathway	0.01957	76
Wnt signaling pathway	0.02192	32
Insulin signaling pathway	0.02287	37
Prolactin signaling pathway	0.02917	20
Central carbon metabolism in cancer	0.03016	19
HIF-1 signaling pathway	0.03100	29
Type II diabetes mellitus	0.03128	15
RNA degradation	0.03765	23
Chronic myeloid leukemia	0.04013	21
Neurotrophin signaling pathway	0.04126	33
Signaling pathways regulating pluripotency of stem cells	0.04285	36
Pathogenic Escherichia coli infection	0.04285	17

Table S1: A list of KEGG pathways found as significantly associated with five miRNAs: miR-375, miR-582-3p, miR-326, miR-181c-5p, and miR-99a-5p. The analysis was performed using DIANA-miRPath v3.0 [18].

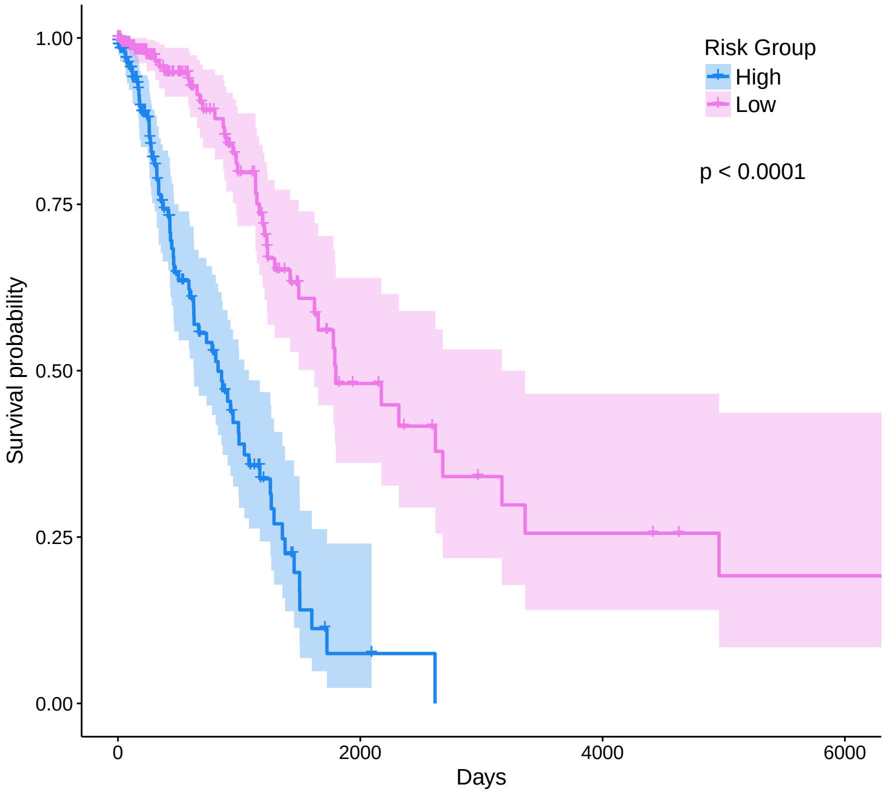

Figure S1: Estimated survival curves for high and low risk groups based on the entire dataset (i.e., combining both Model and Test sets). The p-value provided correspond to the log-rank test. The colored region represents the 95% point-wise CIs.

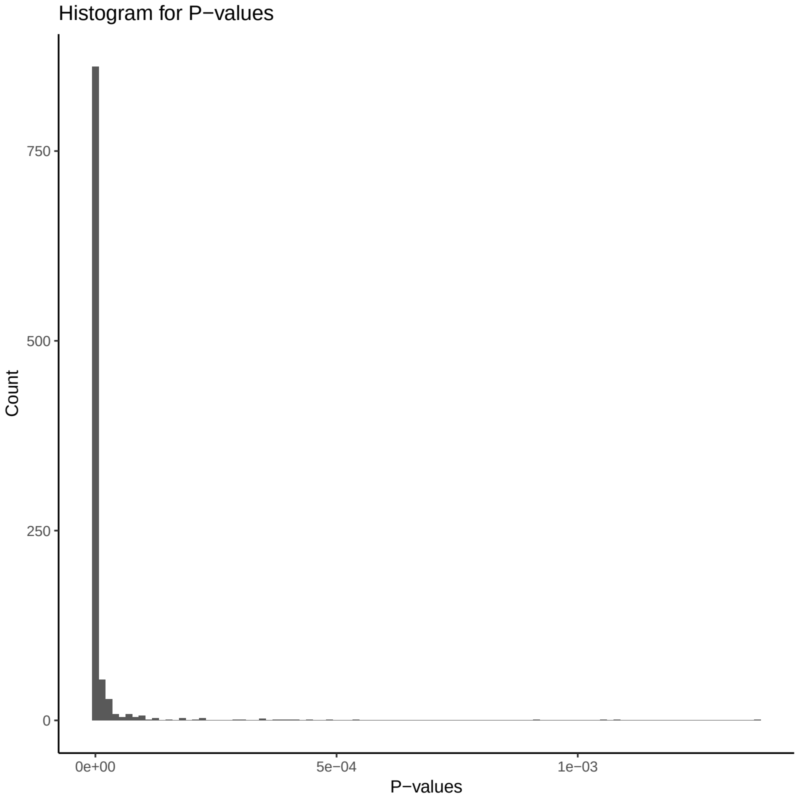

Figure S2: A histogram produced for the distribution of p-values that were calculated for the likelihood ratio test, which compares the Cox regression model that contained only cancer stage and age effects versus the model contained the effects of five miRNAs (miR-375, miR-562-3p, miR-326, miR-181-5p, and miR-99a-5p), in addition to cancer stage, and age. P-values were obtained using 1000 randomly selected folds of size n =320 of the original data.

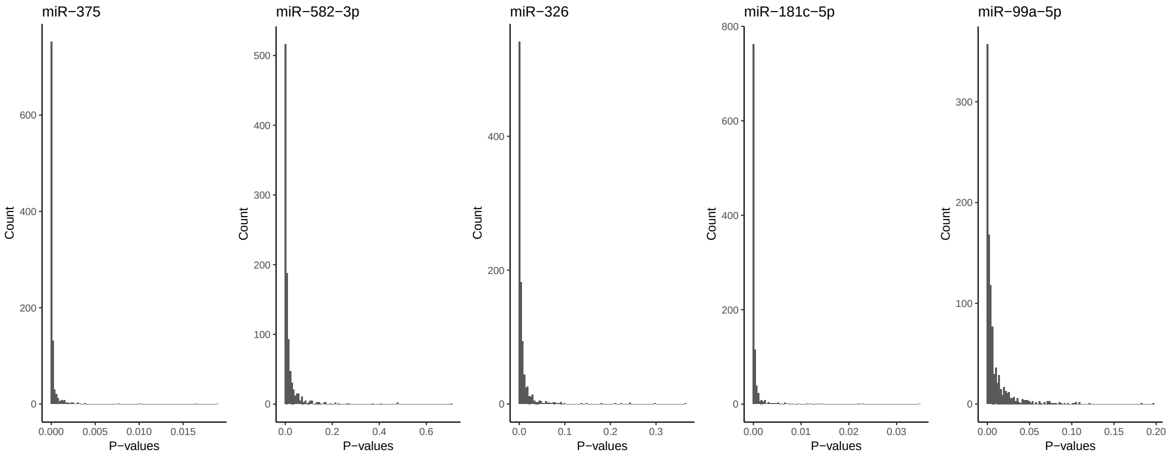

Figure S3: Histograms produced for distributions of p-values that were calculated for the likelihood ratio test, which compares the Cox regression model that contained only cancer stage and age effects versus models contained effects of the each five individual miRNAs (miR-375, miR-562-3p, miR-326, miR-181-5p, and miR-99a-5p), in addition to cancer stage, and age. P-values were obtained using 1000 randomly selected folds of size n =320 of the original data.
